# Study on the influence mechanism of students’ behavior of participation in industrial colleges—Analysis framework based on theory of planned behavior

**DOI:** 10.3389/fpsyg.2022.1037536

**Published:** 2022-11-24

**Authors:** Yuanbao Zhang, Xiangdong Shen, Jinyu Song, Tiantian Huang

**Affiliations:** ^1^School of Business, Changshu Institute of Technology, Changshu, China; ^2^School of Electronic and Information Engineering, Changshu Institute of Technology, Changshu, China

**Keywords:** industrial college, college student, influence mechanism, theory of planned behavior, students’ behavior

## Abstract

As a new organizational form for the coordinated development of education and industry, industrial college is an important carrier for application-oriented colleges and universities to implement application-oriented talent training. Based on Theory of Planned Behavior (TPB) as a theoretical framework, this research constructs a model of the influencing factors of college students’ participating in industrial colleges from five criteria: behavioral attitudes, subjective norms, perceived behavioral control, behavioral intentions and situational factors. In this study, participants (*N* = 541) have completed a questionnaire to assess their behavior of participation in the industrial colleges by using the structural equation model (SEM). It turns out that perceived needs, mandatory norms, exemplary norms, and self-efficacy significantly positively affect college students’ intention to participate in industrial colleges. Behavior intention and self-efficacy significantly positively affect college students’ participation in industrial colleges. Behavior intentions act as a complete mediator between perceived needs and behavior, as well as between exemplary norms and behavior. Behavioral intentions partially mediate between mandatory norms and behavior, as well as between self-efficacy and behavior. In addition, school support plays a significant positive moderating role in college students’ intention and behavior of participating in industrial colleges. These findings not only expand the relevant literature on college students’ learning behavior, but also provide useful enlightenment for college education managers on how to stimulate college students’ endogenous motivation to participate in industrial colleges.

## Introduction

Industrial College is a new organizational form of integration of industry and education for colleges and universities, which is guided by serving specific industrial clusters, guided by docking industrial technological innovation, supported by gathering innovative resources, and with the fundamental goal of cultivating high-quality talents. It is an entity collaborative education platform jointly built, managed, and shared by colleges and universities, local governments, industries, leading enterprises, and other entities (Huang, 2021). Practice shows that industrial college emphasizes the integrated development of talent training, scientific research, technological innovation, and enterprise service and student entrepreneurship. It breaks the traditional closed school running mode of colleges and effectively integrates the school running resources of enterprises, industries, and local governments. It has become an important school running mode of application-oriented colleges and universities (Shen, 2021). The Chinese government’s Guide to the Construction of Modern Industrial Colleges (for Trial Implementation) points out that modern industrial colleges are the important platform to promote the “connection between curriculum content and technological development, the connection between teaching process and production process, and the integration of talent training and industrial demand.” It is an important way to realize the organic connection of industrial chain, innovation chain and education chain; it makes a significant contribution to optimize the knowledge frame and ability structure of talents. For example, Industrial College of Artificial Intelligence of Nanjing University of Information Science and Technology, Industrial college of Intelligent Manufacturing Equipment of Yangzhou University, Aliyun School of Big Data of Changzhou University and Photovoltaic Technology Industrial College of Changshu Institute of Technology have become important carriers for the cultivation of high-quality applied, compound, and innovative talents.

The industrial college of Chinese universities mainly adopts the directional recruitment mode, that is, the college students who have entered the University voluntarily apply according to their personal interests and hobbies, and enter the corresponding industrial college after selection. For example, the “2.5 + 0.5 + 1” mode adopted by most colleges and universities at present, that is, in the first 2.5 years after enrollment, students complete general courses, professional basic courses and compulsory courses in their major. From the 6th semester, students can apply to enter the specific “project class” of the industrial college according to their personal interests to study the school enterprise cooperation course for a period of 0.5 academic years. In the last year, he will enter the cooperative enterprise and complete the training links such as enterprise courses, centralized practice, on-the-job training and graduation thesis. Although the first mock exam is regarded as one of the most effective ways to train applied talents, it has been widely recognized in the theoretical and practical circles. However, in the process of practice, we found that students’ intention to apply to join industrial colleges is not strong, and some industrial colleges even fail to set up “project classes” due to too few applicants. Therefore, there is an urgent need to analyze the primary factors affecting students’ behavior in industrial colleges and their action mechanism based on students’ behavior intention.

Compared with the discipline talent training mode, the industry college training mode breaks the inherent combination mode of teaching materials, courses, teachers, classrooms, and academic assessment in the traditional talent training scheme. College students are very different in the choice between the traditional familiar mode and the new unfamiliar mode (Yi, 2014; Chen and Fang, 2021). This difference first comes from the individual’s evaluation of the costs, benefits, needs and risks generated by the two models. Rational individuals always seek advantages and avoid disadvantages, and often repeatedly compare needs with costs, risks, and benefits, so as to make the best choice (Zu et al., 2013; Milsom and Coughlin, 2015). Secondly, college students’ behavior decisions will be influenced by schools, teachers, parents, seniors, classmates, and important others. For example, through the study of 4,853 college students’ behavior of re-choosing majors in China research universities, Xiong et al. (2019) found that school policy orientation, teachers’ attitude, parents’ education level, and the actual behavior of seniors, classmates and important others will have a significant impact on college students’ intention to change their majors. Thirdly, college students’ behavior decision-making will be affected by individual self-efficacy and resource control. For example, Zhang and Chen (2021) found that self-efficacy, active personality, career adaptability, career self-confidence and entrepreneurial experience have a significant impact on college students’ entrepreneurial behavior through the study of 343 college students’ entrepreneurial behavior in Guangdong Province, China. Because there are various factors affecting college students’ behavior decision-making, a fundamental question is what factors drive or block college students’ academic behavior decision-making. With the answer, the following potential actions can be taken to enable to enhance the enthusiasm of college students to participate in industrial colleges. Therefore, Ajzen’s TPB model is used as our theoretical frame (Ajzen, 1991).

Theory of Planned Behavior (TPB) is main theoretical basis for interpreting and predicting human behavior in social psychology. It is widely used in social psychology to interpret and predict different human behaviors (Botetzagias et al., 2015; Echegaray and Hansstein, 2017; Taufique and Vaithianathan, 2018). In the past, Theory of Planned Behavior (TPB) was used to study college students’ behavior, focusing on their consumption behavior, extracurricular activity behavior and entrepreneurial behavior (Seo et al., 2007; Hu, 2014; Lee and Kim, 2019; Zhang and Chen, 2021; Yu et al., 2022). The results show that behavior attitude, subjective norms, perceived behavior control and behavior intention significantly affect college students’ behavior decision-making. Although these studies used various determinant models to determine the behavior of college students, most studies did not distinguish between educational situational factors, such as school behavior or students’ spontaneous behavior, formal educational behavior, or informal educational behavior. Sutton (1998) pointed out that behavior attitude, subjective norms, and perceived behavior control explain behavior intention (40%–50%), which is much higher than the actual behavior of the individual (19%–38%). That is, situational adjustment variables may exist between behavior intention and behavior of college students. In this case, the present literature using TPB model to investigate college students’ behavior has limited interpretation of college students’ behavior. In order to make up for the theoretical blankness, based on the TPB model, this study takes the educational situational factors as the regulating variables between college students’ behavior intention and behavior, and tests the predictors of college students’ participation in industrial colleges from six aspects: subjective norms, behavior attitude, behavior intention, perceived behavior control, and educational situation.

In a word, the purpose of this study is to integrate the perspective of TPB model, combine the talent training mode of industrial needs, and explore the antecedents and regulation mechanism of college students’ participation in industrial colleges. In detail, this study is going to answer: (1) will subjective norms, behavior attitude, and perceived behavior control affect college students’ intention to participate in industrial colleges? (2) Will behavior intention and perceived behavior control affect college students’ behavior of participating in industrial college? (3) Is there a mediating effect between subjective norms, behavior attitude, perceived behavior control and college students’ behavior? (4) Do educational situational factors have a moderating effect on college students’ intention and behavior to participate in industrial colleges? The keys to these questions will contribute to rationally explain the forming mechanism of college students’ behavior of participating in industrial colleges, and then continuously improve the training mode of applied talents.

## Theoretical model and hypothesis development

### Theoretical model

**Figure 1:**
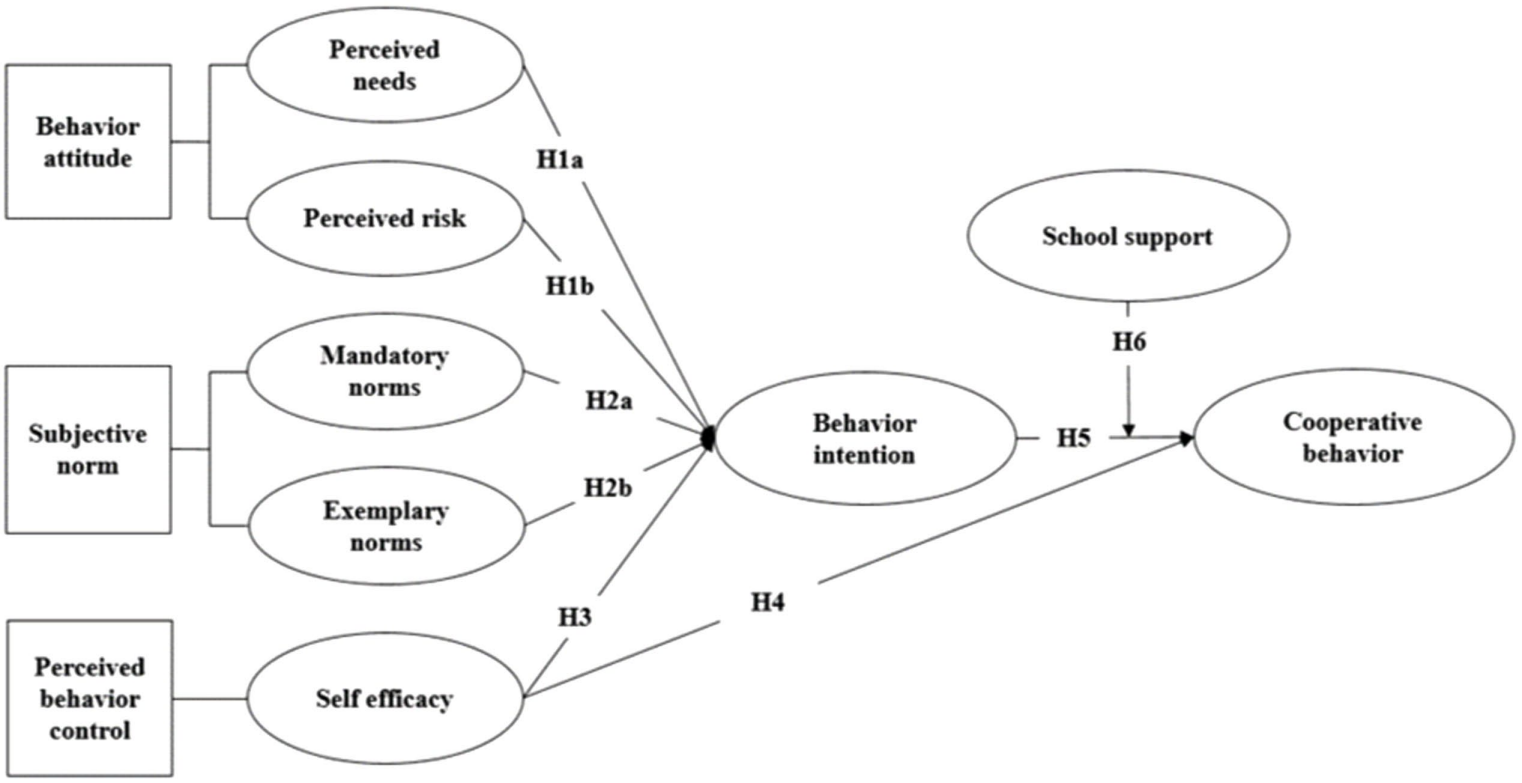
Research model.

As maturing individuals, college students generally have a certain rational thinking about their academic behavior. By systematic collection, analysis, and utilization of related information, they can fully consider the implementation of a particular behavior and the impact of the behavior after it occurs (Tian, 2018). Generally speaking, college students’ participation in industrial colleges will be affected by many factors: individual learning needs, unknown risks and expected benefits, schools, teachers and parents, senior students, classmates and important others, self-learning efficacy and control (Liu, 2021). From the perspective of educational administrators, it is imperative to have a clear understanding of what factors influence students’ behavior in attending industrial colleges. Combined with the training law of applied talents and present research results, college students’ behavior attitude, perceived behavior control, subjective norms, and educational situational factors will affect college students’ academic behavior decision-making (Saraih et al., 2020). Therefore, the following models are proposed (Figure 1).

### Hypothesis development

#### Behavior attitude and behavior intention

Behavior attitude is the basic position of the behavior subject on the implementation of a specific behavior (Ajzen, 2002). It is the internal mechanism of the formation of behavior intention. Generally speaking, before the behavior decision-making, the rational behavior subject will always make a subjective evaluation of the behavior result in advance. When he has a positive attitude toward the behavior result, he will be ready to pay more time and vigor for it (Mahagaonkar, 2010). Ran (2021) believes that behavior attitude can be measured by two indicators: Perceived needs and perceived risk of behavior subjects. Specifically, perceived needs positively affect behavior intention, and perceived risk negatively affects behavior intention. The perceived needs of college students to participate in industrial colleges mainly come from the demand of skill learning, career development and high-quality employment. Perceived risk mainly comes from the uncertainty of academic performance caused by the change of school enterprise curriculum connection, educational environment, and assessment methods. Hence this study puts forward the hypothesis as follows:

*H1a*: Perceived needs have positive effects on college students’ intention to participate in industrial colleges.

*H1b*: Perceived risk has a negative effect on college students’ intention to participate in industrial colleges.

#### Subjective norms and behavior intention

Subjective norms refer to the outside forces that the behavior subject feels for participating in or not participating in a specific behavior, especially the pressure brought by various social relations in the social structure. Generally speaking, behavior subject with more positive external evaluation and higher support may have stronger behavior intention (Conner et al., 2007). Due to the existence of normative consciousness, college students’ behavior will be affected by the attitudes of schools, teachers, parents, and peers to a certain extent in addition to their personal preferences. Cialdini et al. (1991) believes that individual subjective norms can be measured by two indicators: mandatory norms and exemplary norms. Specifically, mandatory norms are mainly reflected in the attitude of schools, teachers, and parents toward college students’ participation in industrial colleges. The higher their evaluation is, the stronger college students’ intention to participate is. Exemplary norms are mainly manifested in the demonstration effect brought by peer groups, especially when peer groups have achieved certain results by participating in industrial colleges, the more obvious this demonstration effect is. Hence this study puts forward the hypothesis as follows:

*H2a*: Mandatory norms have positive effects on college students’ intention to participate in industrial colleges.

*H2b*: Exemplary norms have positive impact on intention of college students’ participation in industrial colleges.

#### Perceived behavior control and behavior intention

Perceived behavior control is the difficulty degree that behavior subject feels to carry out a certain activity. It is the unconscious complete control behavior of an individual, consistent with the self-efficacy proposed by Ajzen (1991). They all emphasize the degree of control over a specific behavior, that is, the ability judgment and confidence of the behavior subject on whether it can successfully achieve the expected goal (Bandura, 1987). The self-efficacy of college students participating in industrial colleges is mainly manifested in their subjective perception of the ability to adapt to the new education model and successfully complete their studies. The more confident college students are about this ability, the easier it is to stimulate their strong intention to participate in industrial colleges. For example, Jiang (2016) conducted an empirical study on 293 college students and found that college students’ self-efficacy significantly positively affects their willingness to use WeChat mobile learning. Hence this study puts forward the hypothesis as follows:

*H3*: Self-efficacy has a positive effect on college students’ intention of participation in industrial colleges.

#### Perceived behavior control and cooperative behavior

As mentioned above, the perceived behavior control of college students participating in industrial colleges can be measured by self-efficacy. Ajzen and Sexton (1999) pointed out that self-efficacy directly affects both individuals’ behavior intention and actual behavior. Especially when the predicted behavior is not completely controlled by consciousness, self-efficacy can have a direct effect on individual behavior. For example, Lu and Zhang (2021) studied 337 college students’ knowledge payment behavior and found that self-efficacy had a significantly direct effect on college students’ knowledge payment behavior. Hence this study proposes hypothesis as follows:

*H4*: Self-efficacy has a direct effect on college students’ behavior of participating in industrial colleges.

#### Behavior intention and cooperative behavior

Behavior intention is behavior subject’s subjective probability and willingness to put in the effort when performing a particular act (Elliott et al., 2007). Previous studies have shown that behavior intention is highly connected with behavior, which shows that behavior intention has a positive effect on individual behavior and has a good predictive effect on individual actual behavior. For example, Hu (2021) conducted an empirical study on 299 college students’ online learning behavior and found that behavior intention has a remarkable positive effect on online learning behavior, and college students’ behavior with strong intention is more prominent. The behavior of college students participating in industrial college will be affected by individual behavior intention to a certain extent, including learning time investment, energy investment and academic effort. Hence this study puts forward the hypothesis as follows:

*H5*: Behavior intention has a positive effect on college students’ behavior of participating in industrial colleges.

#### Situational factors, behavior intention, and cooperative behavior

The relationship between behavior intention and behavior is not just simple linearity. The explanation of behavior intention to individual actual behavior is only 12%–38%, indicating that there are other variables between them, which promote or hinder the occurrence of individual behavior through regulation (Sutton, 1998). For example, the empirical study of 403 Chinese college students’ entrepreneurial behavior by Zhang (2020) found that environmental support has a significant moderating effect on college students’ entrepreneurial intention and behavior, especially the support from the government and stakeholders, which is easier to stimulate college students’ entrepreneurial behavior. The situational factors of college students’ participation in industrial colleges mainly come from the school’s educational environment, including the school’s attention, policy guarantee and publicity (Zhang et al., 2018; Zhang et al., 2020). Therefore, this study puts forward the hypothesis as follows:

*H6*: School support has a positive moderating effect on college students’ intention and behavior of participating in industrial colleges.

## Research methodology

### Instrument

In order to guarantee that the questionnaire was correct and effective, all the measurement items are obtained from the available literature after appropriate modification based on the research objective and the actual state of the research object. Specifically, the project for measuring college students’ perceived needs is adapted from Zhang (2015), the project for measuring college students’ perceived risk is adapted from Deng (2021), the project for measuring college students’ mandatory norms and exemplary norms is adapted from Gu et al. (2018), and the project for measuring college students’ self-efficacy is adapted from Peng et al. (2019), The project for measuring college students’ behavior intention is based on Feng and Zhang (2020), and the project for measuring college students’ behavior and situational factors is adapted from Liu (2021). The final questionnaire is provided in Appendix A. All items were measured by the 5-point Likert scale, ranging from 1 (very disagree) to 5 (very agree).

### Data collection

In this study, 541 college students from Industrial College of E-commerce, Industrial College of Photovoltaic Technology and Industrial College of Elevator Engineering of Changshu Institute of Technology were selected as the survey objects. The reasons for choosing the industrial colleges of Changshu Institute of Technology are as follows: Changshu Institute of Technology is a domestic application-oriented undergraduate industry education integration development project construction university, and a pilot University for the training of outstanding engineers of the Ministry of education. It has successively established many modern industrial colleges, such as photovoltaic technology, elevator engineering, automobile engineering, textile and clothing, artificial intelligence, pharmaceutical biotechnology, intelligent manufacturing, e-commerce and so on, and initially formed an applied talent training mode relying on the industrial colleges. Among them, the Industrial College of Photovoltaic Technology, established in 2009, is the first entity school running institution of photovoltaic industry in Jiangsu Province; The Industrial College of Elevator Engineering officially enrolled students in 2012 and is the first school running institution of elevator industry entity in China; Founded in 2021, the Industrial College of E-commerce is a modern industry college with Changshu local characteristics. In general, the three industrial colleges are well representative. The data collection of this study was conducted in January 2022. The questionnaire was released on China’s largest online questionnaire platform, namely, sojump, and college students from the Industrial College of E-commerce, the Industrial College of Photovoltaic Technology and the Industrial College of Elevator Engineering were invited to fill in (Table 1).

**Table 1:** Demographics of the survey respondents (*N* = 541).

Demographic	Category	Frequency	%
Gender	Male	216	39.9
	Female	325	60.1
Professional types	Liberal arts	304	56.2
	Science and engineering	237	43.8
Industrial College type	Electrical business class	171	31.6
	Photovoltaic technology class	186	34.4
	Elevator engineering class	184	34.0

## Data analysis and results

### Reliability and validity

CFA further tested the reliability and effectiveness of the structure. As Table 2 shows, the Cronbach’s a, and composite reliability (CR) values of each structure are between 0.822 and 0.948, higher than the recommended threshold of 0.7 (Straub et al., 2004), showing a satisfactory reliability level. For construct validity, both convergent and discriminant validity will be checked. Convergent validity is verified by examining the Average Variance Extracted (AVE) and exponential load. As Table 2 shows, all AVE values were above the recommended level of 0.5 (Fornell and Larcker, 1981). The standard loadings for all items were above the required threshold of 0.7, with a significance of 0.001. This illustrated fine convergent validity (Chin et al., 1997).

**Table 2:** Results of confirmatory factor analysis.

Construct	Indicator	Standard loading^a^	Cronbach’s α	CR	AVE
Perceived needs	PEN1	0.754	0.822	0.822	0606
	PEN2	0.796			
	PEN3	0.785			
Perceived risk	PER1	0.826	0.889	0.890	0.729
	PER2	0.849			
	PER3	0.886			
Mandatory norms	MAN1	0.802	0.907	0.912	0.776
	MAN2	0.919			
	MAN3	0.917			
Exemplary norms	EXN1	0.884	0.930	0.933	0.823
	EXN2	0.946			
	EXN3	0.890			
Self-efficacy	SEE1	0.828	0.911	0.912	0.776
	SEE2	0.897			
	SEE3	0.915			
Behavior intention	BEI1	0.910	0.947	0.948	0.858
	BEI2	0.939			
	BEI3	0.929			
Cooperative behavior	COB1	0.937	0.938	0.939	0.838
	COB2	0.920			
	COB3	0.888			
School support	SCU1	0.942	0.945	0.946	0.855
	SCU2	0.938			
	SCU3	0.892			

χ2 = 3.020, CFI = 0.967, TLI = 0.959, GFI = 0.908, NFI = 0.951, RMSEA = 0.061.

a All standard loadings were significant at *p* < 0.001.

Discriminant validity was assessed by comparing the square root of AVE and the correlation value. The discriminant validity was evaluated by comparing the square root of AVE for each construct with the correlations between that construct and other constructs (Fornell and Larcker, 1981). As Table 3 illustrated, the square roots of the AVEs (i.e., diagonal elements) were greater than the correlation between structures described in non-diagonal entries, so it shows that the discriminant validity is sufficient.

**Table 3:** Results of discriminant validity testing.

	Mean	S.D.	PEN	PER	MAN	EXN	SEE	BEI	COB	SCU
PEN	4.289	0.875	**0.778**							
PER	3.862	0.943	0.701	**0.854**						
MAN	3.948	0.967	0.622	0.572	**0.881**					
EXN	3.948	1.002	0.616	0.586	0.805	**0.907**				
SEE	3.947	0.902	0.647	0.533	0.739	0.665	**0.881**			
BEI	4.060	0.906	0.675	0.507	0.714	0.701	0.806	**0.926**		
COB	4.023	0.915	0.640	0.510	0.724	0.672	0.820	0.920	**0.915**	
SCU	4.057	0.899	0.628	0.506	0.725	0.644	0.768	0.835	0.882	**0.925**

PEN, Perceived needs; PER, Perceived risk; MAN, Mandatory norms; EXN, Exemplary norms; SEE, Self-efficacy; BEI, behavior intention; COB, Cooperative behavior; SCU, School support. Diagonal bold entries are square root of AVE; All others are correlations coefficients.

Since the data was based on self-report from single origin, we assessed common methodological bias by performing a statistical analysis. Firstly, we made further efforts to assess the method factors on the basis of the procedure proposed by Liang et al. (2007). The results show that the loads of main variables were significant at the level of *p* < 0.001, while the factor loads of common methods were not significant. These results suggest that common method bias was not likely to be the focus of this study.

Second, we performed a multicollinearity test to examine the correlation between independent variables. A variance expansion factor (VIF) value above 10 indicate multicollinearity issues. As shown in Table 4, the VIF values of variables in this study were below 10, suggesting the absence of multicollinearity.

**Table 4:** Results of multicollinearity analysis.

Model	Unstandardized coefficient		Standardized coefficient	*t*	Significance	Multicollinearity statistics	
	B	Standard error	β			Tolerance	VIF
1(con.)^a^	0.490	0.099		0.497	0.620		
PEN	−0.260	0.030	−0.022	−0.863	0.388	0.501	1.998
PER	0.120	0.025	0.012	0.488	0.626	0.561	1.783
MAN	0.290	0.031	0.030	0.918	0.359	0.322	3.107
EXN	0.010	0.028	0.001	0.035	0.972	0.352	2.844
SEE	0.149	0.032	0.143	4.612	0.000	0.347	2.881
BEI	0.486	0.036	0.485	13.652	0.000	0.266	3.754
SCU	0.335	0.033	0.331	10.127	0.000	0.314	3.185

a Dependent variable: Cooperative behavior. PEN, Perceived needs; PER, Perceived risk; MAN, Mandatory norms; EXN, Exemplary norms; SEE, Self-efficacy; BEI, behavior intention; SCU, School support.

### Hypothesis testing

**Figure 2:**
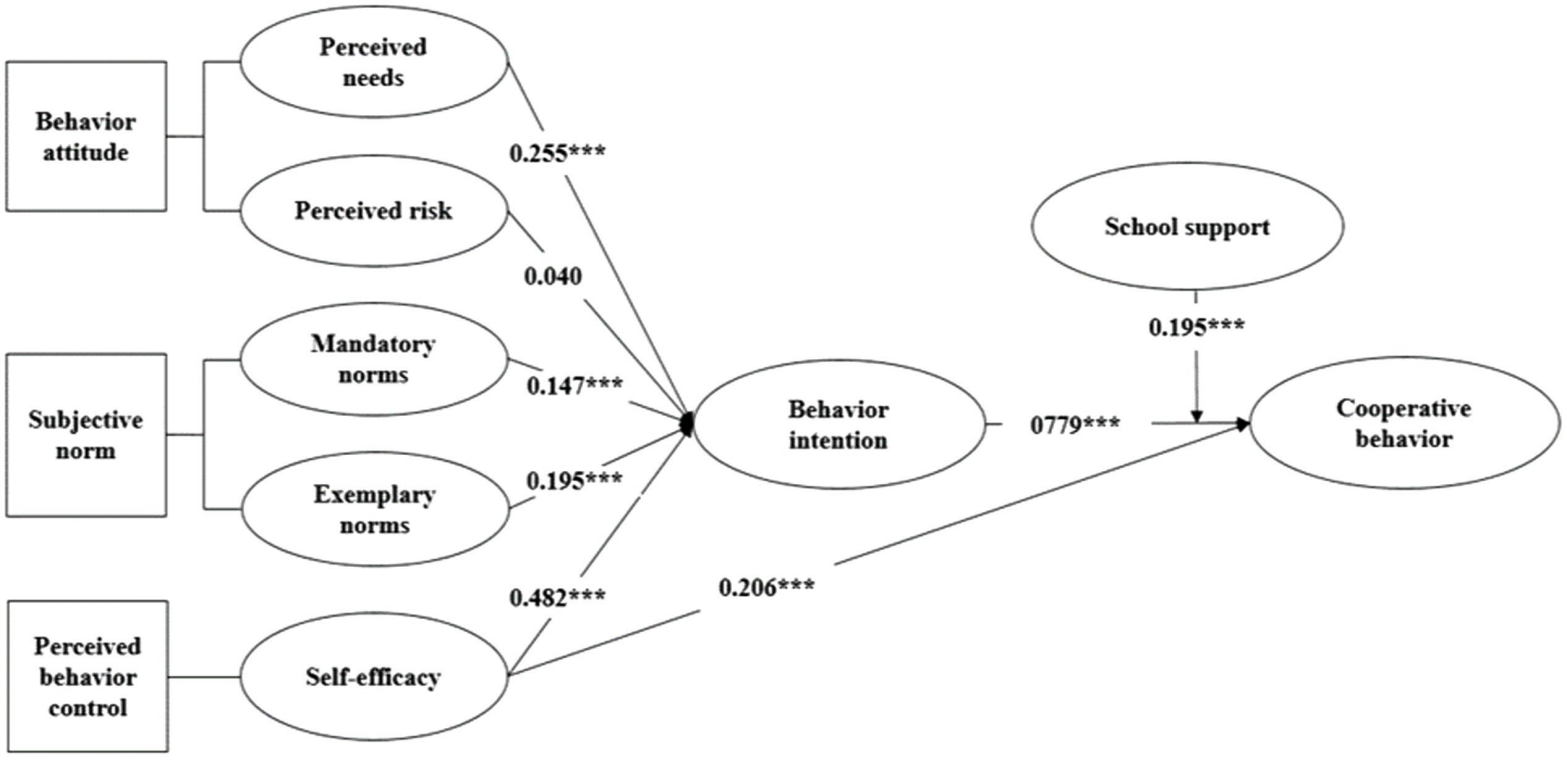
The results of the research model. ^***^p < 0.001.

Figure 2 points out that: perceived needs, mandatory norms, exemplary norms, and self-efficacy have a remarkable positive impact on college students’ intention of participation in industrial colleges, thus supporting hypothesis H1a, H2a, H2b, and H3. Behavior intention and self-efficacy play a markedly positive role in college students’ behavior of participating in industrial college, which supports hypothesis H4 and H5. Perceived risk has no significant effect on college students’ intention to participate in industrial colleges, so they reject hypothesis H1b. This may be because in the cooperation between the school and the local government, the school teachers are mainly responsible for negotiating cooperation with the local government. Therefore, teachers perceive more risks, while students perceive less risks. College students’ intention and behavior of participation in industrial colleges are positively regulated by school support (β = 0.195, *p* < 0.001; Table 5), so as to support H6. Self-efficacy is the biggest influence on college students’ intention to participate in industrial colleges (β = 0.482, *p* < 0.001) among the four antecedents of behavior intention, next is perceived needs (β = 0.255, *p* < 0.001). Behavior intention is the biggest influence on college students’ behavior of participating in industrial college (β = 0.779, *p* < 0.001) among the two antecedents of behavior, next is elf-efficacy (β = 0.206, *p* < 0.001; Table 6).

**Table 5:** Moderation variable analysis.

Adjustment variable path	Estimate	S.E.	C.R.	*P*-value
Behavior intention → cooperative behavior	−0.140	0.111	−1.263	0.207^n.s.^
School support → cooperative behavior	−0.431	0.117	−3.670	***
Behavior intention* school support → cooperative behavior	0.195	0.027	7.161	***

*** *p* < 0.001.

n.s., not significance.

**Table 6:** Hypotheses test.

Hypothesis path	Path coefficient	S.E.	*t*-value	*p*-value	Results
H1a: Perceived needs → behavior intention	0.255	0.036	7.012	***	Supported
H1b: Perceived risk → behavior intention	0.040	0.027	1.495	0.135^n.s.^	Unsupported
H2a: Mandatory norms → behavior intention	0.147	0.026	5.699	***	Supported
H2b: Exemplary norms → behavior intention	0.195	0.026	7.481	***	Supported
H3: Self-efficacy → behavior intention	0.482	0.030	15.939	***	Supported
H4: Self-efficacy → cooperative behavior	0.206	0.029	7.024	***	Supported
H5: Behavior intention → cooperative behavior	0.779	0.043	18.280	***	Supported

*** *p* < 0.001.

n.s., not significance.

Then we used the guidance method proposed by the Preacher and Hayes (2008) to test the intermediary effect of trust and satisfaction with the platform. The application and inspection of mesomeric effect is the principal trend of management studies. As Table 7 is shown, behavior intentions are fully mediated between perceived needs and behavior, as well as between exemplary norms and behavior; Behavior intentions are partially mediated between mandatory norms and behavior, as well as between self-efficacy and behavior; with a bootstrap confidence interval of 95%, excluding zero. This finding suggests that behavior intention mediates among perceived needs, mandatory norms, exemplary norms, self-efficacy of college students’ behavior of participation in industrial colleges.

**Table 7:** Results of mediating effect analysis.

IV	M	DV	IV → M	IV → DV	M → DV	Indirect effect	CIs	Mediation
PEN	BEI	COB	0.676^***^(0.056)	0.040 (0.043)	0.893^***^(0.040)	0.603^**^(0.043)	[0.535, 0.670]	Yes
MAN			0.712^***^(0.036)	0.139^***^(0.033)	0.821^***^(0.039)	0.584^*^(0.330)	[0.502, 0.651]	Yes
EXN			0.695^***^(0.037)	0.052 (0.032)	0.883^***^(0.039)	0.614^**^(0.032)	[0.542, 0.702]	Yes
SEE			0.806^***^(0.036)	0.225^***^(0.042)	0.738^***^(0.044)	0.595^*^(0.042)	[0.502, 0.692]	Yes

95% Bootstrap CIs for the indirect effect.

IV, independent variable; M, mediator variable; DV, dependent variable; PEN, Perceived needs; MAN, Mandatory norms; EXN, Exemplary norms; SEE, Self-efficacy; BEI, behavior intention; COB, Cooperative behavior. IV → DV is significant (M not included in the model); IV → M is significant; M → DV is significant (or the meaningful reduction in effect) of the relationships between the initial IV and DV in the presence of mediator. SEs in brackets.

* Significance at *p* < 0.05.

** Significance at *p* < 0.01.

*** Significance at *p* < 0.001.

## Discussion and implications

### Discussion of findings

Interesting findings emerged from this study. The results reflect that perceived needs, mandatory norms, exemplary norms, and self-efficacy have certain extend influence on college students’ intention and behavior to participate in industrial colleges. Perceived needs have stronger effects on behavior intention (β = 0.255, *p* < 0.001), Mandatory norms have stronger effects on behavior intention (β = 0.147, *p* < 0.001), Exemplary norms have stronger effects on behavior intention (β = 0.195, *p* < 0.001), and Self-efficacy has greater influence on behavior intention (β = 0.482, *p* < 0.001). In addition, perceived risk has no significant effect on college students’ intention to participate in industrial colleges. This might occur because college students live in an arranged university education system for a long time. They believe that the teaching arrangement of the school is reasonable, scientific, and conducive to the development of students, and lack enough attention to the transformation of education mode and the resulting potential academic risks. The results are in line with those of earlier studies (Liu, 2021); it shows that college students’ intention to participate in industrial colleges is principally affected by perceived needs, mandatory norms, exemplary norms and self-efficacy.

Secondly, behavior intention and self-efficacy have prominent positive effect on college students’ behavior of participating in industrial colleges. Concerning behavior intention and Self-efficacy’s influence on cooperation behavior, our findings suggest that behavior intention and self-efficacy can foresee cooperation behavior and that behavior intention (β = 0.779, *p* < 0.001) has more important decision-making influence on cooperative behavior than self-efficacy (β =0.206, *p* < 0.01). This indicates that behavior intention is the leading factor affecting college students’ behavior of participating in industrial colleges. In addition, we also find that behavior intention fully mediate between perceived needs and behavior, as well as between exemplary norms and behavior; Behavior intentions partially mediate between mandatory norms and behavior, as well as between self-efficacy and behavior. Our findings extend those of previous studies (Hu, 2021), indicating that behavior intention and self-efficacy have the similar influence on college students’ learning style choice. Ultimately, the results demonstrated that school support can effectively adjust the college students’ intention and behavior to participate in industrial colleges. The interaction of school support and intention is positively related to college students’ participation in industrial colleges (β = 0.195, *p* < 0.01). The hypothesis is validated and aligned with the literature, highlighting the regulation of education environment on college students’ behavior intention and behavior (Zhang, 2020; Jiang, 2021).

### Theoretical contribution

This study illustrates more clearly the formation mechanism of college students’ participation in industrial colleges through TPB model. Many studies use TPB model to explore the impact of college students’ subjective perception on academic behavior (Abdullah et al., 2021; Jiang, 2021). These studies suggest that college students’ subjective norms, behavior attitude, and perceived behavior control positively affect learning behavior. However, most of these studies focus on the main factors affecting college students’ behavior from the individual psychological level (Peng et al., 2019; Liu, 2021), but lack of attention to the situation when college students’ behavior occurs. Unlike former studies, besides personal subjective cognitive factors, situational factors of school support also affect college students’ academic decision behavior heavily.

According to TPB model, we identified six primary factors affecting college students’ behavior of participating in industrial colleges, namely perceived needs, mandatory norms, exemplary norms, self-efficacy, behavior intention and school support. Specifically, perceived needs, mandatory norms, exemplary norms, and self-efficacy significantly affect college students’ participation in industrial colleges through behavior intention. Behavior intention has intermediary effect between perceived needs, exemplary norms, mandatory norms, self-efficacy, and college students’ actual behavior. Behavior intention and self-efficacy affect college students’ participating in industrial colleges markedly. In addition, school supports positively regulate college students’ intention and behavior of participating in industrial colleges. This study complements the academic literature of college students’ learning behavior mechanism; it also gives a renew angle for college education managers to assess the talent training model of industrial colleges.

### Managerial implications

This study offer helpful managerial implications from two areas. Firstly, as the knowledge production mode changes, college students’ knowledge structure and ability structure are more and more rooted in application scenarios and interdisciplinary environment, which objectively extends talent training from the interior of universities to multiple subjects such as enterprises and scientific research institutes. As an important carrier of applied talent training, how to attract more college students to study in industrial colleges has become the focus of talent training mode reform in applied universities. This study found that perceived needs, mandatory norms, exemplary norms, self-efficacy, and behavior intention significantly positively affect college students’ behavior of participating in industrial colleges, and then revealed the internal mechanism of college students’ behavior of participating in industrial colleges. For college education administrators, it is necessary to strengthen the publicity of the talent training mode of the industrial colleges, including the curriculum, application process, teaching mode, assessment requirements and future career development of the industrial colleges, so as to improve the rational cognition of college students and stimulate the endogenous motivation of college students to participate in the industrial colleges. On the other hand, we should stimulate college students’ self-efficacy and turn their intention to participate into practical action with the help of on-the-spot observation, immersive education, successful cases and example strength.

Secondly, the talent training mode of industrial colleges is essentially a “top-down” school education reform behavior. School policy support is an important guarantee to improve the quality of cooperative education between colleges and enterprises. It was found that school support significantly regulate college students’ intention and behavior of participating in industrial colleges. Hence improving the talent training system of industrial college is another crucial point to enhance the intention of college students to participate in industrial colleges. For application-oriented universities, on the one hand, formulate the connection system between the traditional talent training mode and the talent training mode of industrial colleges to ensure the integration and systematicness of curriculum system, knowledge system and ability system in the process of talent training; On the other hand, formulate the credit mutual recognition and conversion system, and improve the rules, standards, scope and methods of credit mutual recognition and conversion between enterprise courses and school courses, so as to ensure that college students can successfully complete their studies and achieve high-quality employment in the conversion process of the two education modes.

### Limitations and future research

According to TPB model, a behavioral decision-making model of college students participating in industrial colleges was constructed in this study, and the model was tested by the questionnaire data of 541 college students from the Industrial College of E-commerce, the Industrial College of Photovoltaic Technology and the Industrial College of Elevator Engineering of Changshu Institute of Technology. It turned out that the model constructed does a nice job of explaining the problem, but this study still has limitations as follows: firstly, this article chooses application-oriented college students as the object of investigation, which may be different from research universities, colleges and other types of colleges and universities; Secondly, the questionnaire data of this study are all from three industrial colleges of the same university. The total number of samples is relatively thin and the source of samples is relatively single, which may be different from the large sample data. In future research, we can further expand the total number of samples according to the research needs, and take the school type as the regulating variable of college students’ participation in industrial colleges.

## Data availability statement

The original contributions presented in the study are included in the article/supplementary material, further inquiries can be directed to the corresponding author.

## Ethics statement

The studies involving human participants were reviewed and approved by the School of Business, Changshu Institute of Technology. In accordance with national legislation and the institutional requirements, this study does not require written informed consent.

## Author contributions

YZ designed the study and drafted the initial manuscript. YZ, XS, and HT collected the data, performed statistical analysis, and prepared the first draft. JS contributed to the revised manuscript. All authors contributed to the article and approved the submitted version.

## Funding

This study was supported by the 2021 Jiangsu Provincial Social Science Fund Project “Innovative Research on the Integration of Industry and Education in Jiangsu from the Perspective of Symbiosis” (project no. 21JYB008, project leader: YZ).

## Conflict of interest

The authors declare that the research was conducted in the absence of any commercial or financial relationships that could be construed as a potential conflict of interest.

## Publisher’s note

All claims expressed in this article are solely those of the authors and do not necessarily represent those of their affiliated organizations, or those of the publisher, the editors and the reviewers. Any product that may be evaluated in this article, or claim that may be made by its manufacturer, is not guaranteed or endorsed by the publisher.

## Appendix

Appendix A: Questionnaire items.

**Table:**

Construct	Item	Source
Perceived needs	PEN1: College students are facing certain employment pressure	Zhang, 2015
	PEN2: High quality employment needs to enhance practical ability	
	PEN3: I hope to get a stable job in a cooperative enterprise	
Perceived risk	PER1: Facing pressure to adapt to the new teaching model	Deng, 2021
	PER2: Facing the pressure of school-enterprise curriculum connection	
	PER3: Faced with pressure to learn corporate courses	
Mandatory norms	MAN1: My parents supported me to attend the industrial college	Gu et al., 2018
	MAN2: My school supported me to attend the industrial college	
	MAN3: Teachers encourage college students to attend industrial college	
Exemplary norms	EXN1: Senior students actively participate in the study of industrial college	Gu et al., 2018
	EXN2: Classmates actively participate in the study of industrial college	
	EXN3: Significant other actively participate in the study of the industrial college	
Self-efficacy	SEE1: I believe I can fulfill the requirements of industrial college	Peng et al., 2019
	SEE2: I can overcome all kinds of difficulties in the study of industrial college	
	SEE3: I will be able to enter the industrial college smoothly	
Behavior intention	BEI1: I would like to study in an industrial college	Feng and Zhang, 2020
	BEI2: I’m willing to give a lot of time and energy	
	BEI3: I would like to promote the active role of industrial colleges	
Cooperative behavior	COB1: I will actively understand the talent training program of industrial College	Liu, 2021
	COB2: I will ask the teacher about the admission requirements of industrial college	
	COB3: I will apply to the school for admission	
School support	SCU1: School attaches great importance to the training of talents in industrial college	Liu, 2021
	SCU2: School has introduced a policy of mutual recognition of credits	
	SCU3: Our major has actively promoted the industrial College	
